# Honeybees' Distance Perception Changes with Terrain of Flight Path

**DOI:** 10.1371/journal.pbio.0020227

**Published:** 2004-07-13

When a trip for food can require a three-mile flight, it pays to get the directions right, especially if you're a bee. Bees more typically forage within a 600- to 800-yard radius, expending a significant amount of energy—a fact they seem keenly aware of: for reasons that remain unclear, bees tend to ignore directions that send them to a target on water. It's been known since Aristotle's time that returning foragers dance a little jig for their hivemates, presumably regaling them with tales of nectar-laden flora. Some 2,300 years later, zoologist Karl von Frisch correlated dance choreography to the direction and distance of a food source, eventually winning the Nobel prize for his work. Since then, researchers have been working out the details of bee communication, such as how fellow foragers interpret the “waggle dance” and how dancers perceive and convey navigational details of a trip. A key aspect of this information exchange is how bees estimate distance.[Fig pbio-0020227-g001]

**Figure pbio-0020227-g001:**
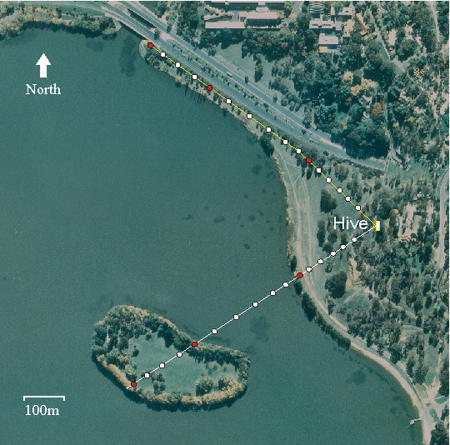
A honeybee's “odometer” generally runs faster when it flies over land than water

Recent studies suggest that bee odometers are driven by the “optic flow” experienced during flight—or, simply put, bees appear to log distance by measuring the rate that images of passing terrain move in their eye during flight. This theory comes from observations that when bees fly a given distance, they indicate a much longer distance—by performing a longer waggle—for low-flying trips than for those at higher altitudes, presumably because flying at higher altitudes limits the bees' ability to perceive changing images. Likewise, bees flying through short, narrow tunnels filled with visual elements waggle a disproportionately long distance. These observations also raise questions about which visual aspects of the environment—contrast, texture, distribution of objects—are most important to a bee's perception of image flow.

To investigate the factors driving the bee trip odometer, Mandyam Srinivasan and colleagues trained bees to feed at locations along two different routes in a natural environment, then compared their waggle dances. One route was entirely over land; the other started on land, shifted to water, and ended back on land. Both routes were the same distance, about 630 yards. Bees trained to feed at a boat in the middle of a lake had no trouble getting there, which was no guarantee based on reports that bees have trouble flying across lakes, often plunging into the drink. They had less success recruiting their colleagues to share in the bounty, even though their waggles clearly placed the feeder on the lake. (This finding supports an earlier, controversial theory that suggests experienced bees know water rarely harbors bee food.)

The length of the bees' waggle dance increased faster with distance when they flew over land than when they flew over water. This disparity indicates that land provides a stronger “odometric signal” than water. “The honeybee's odometer,” the authors explain, “runs at a slower pace when flight is over water.” Overland flights tend to offer high contrasts and rich textures, while flights over water tend to offer low contrasts and sparse textures. Most likely, it is the high contrast of land surfaces that triggers a stronger odometric signal. But land surfaces also show variation in contrast, which was reflected in the bees' dance. One section of the land-only route was a paved bicycle path, a low contrast surface that the bees waggled as a relatively shorter distance.

Whether or not the contrast theory holds, Srinivasan and colleagues conclude, differences in the visual environment trigger differences in odometric signal. The odometer racks up yards depending on the nature of the terrain, whether it be land or water, during flight. The great Belgian playwright and avid bee-keeper Maurice Maeterlinck wondered at the language of bees in his 1901 book, *The Life of the* *Bee*, deciding it must correspond “to senses and properties of matter wholly unknown to ourselves.” As Srinivasan and colleagues show here, the bee's view of the world indeed corresponds to a unique way of interpreting the landscape—and of sharing news of their travels with their hivemates.

